# Fault Diagnosis Method for Electric Vehicle In-Wheel Motor Bearings Based on Improved SVMD and ResNet-KAN

**DOI:** 10.3390/s26113586

**Published:** 2026-06-04

**Authors:** Liang Zhang, Yanlong Xu, Hongtao Xue, Chengchao Zhu, Zhihua Xu

**Affiliations:** 1 School of Automotive and Traffic Engineering, Jiangsu University, Zhenjiang 212013, China; zhangliang@stmail.ujs.edu.cn (L.Z.); xuyanlong@stmail.ujs.edu.cn (Y.X.); 15150852339@163.com (C.Z.); 15852965595@163.com (Z.X.); 2 International Joint Laboratory on Mobility Equipment and Artificial Intelligence for IT Operations, Zhenjiang 212013, China

**Keywords:** in-wheel motor, bearing failure, successive variational mode decomposition, feature extraction, residual neural network, intelligent diagnosis

## Abstract

In-wheel motor bearings in electric vehicles operate in harsh environments where strong background noise often masks early fault features, limiting the accuracy of traditional diagnostic methods. This study proposes an intelligent fault diagnosis framework integrating improved Successive Variational Mode Decomposition (SVMD) with a ResNet–Kolmogorov–Arnold Network (ResNet-KAN). To enhance feature extraction, a multi-strategy Crested Porcupine Optimizer (CPO) is employed to adaptively optimise SVMD parameters. Subsequently, a Gramian angular difference field (GADF) reconstruction strategy transforms one-dimensional vibration signals into two-dimensional images to improve spatial distinguishability. Finally, a ResNet-KAN model, featuring a ReLU-based non-linear classification head, is developed to capture complex fault boundaries more effectively than traditional linear layers. Experimental results demonstrate that the CPO-SVMD method increases the kurtosis of extracted components by at least 25.6% compared to traditional optimisation methods. Furthermore, the ResNet-KAN model achieves an identification accuracy exceeding 98% on the in-wheel motor bearing dataset, outperforming 2DCNN, ResNet, and ViT models by at least 2%. This integrated approach provides a robust, high-precision solution for the intelligent condition monitoring and early warning of in-wheel motor drive systems under complex, high-noise operating conditions.

## 1. Introduction

With the rapid development of the electric vehicle (EV) industry, the in-wheel motor has emerged as a promising solution due to its compact structure, high efficiency, fast response, and independent control capabilities [1]. Electric vehicles employing in-wheel motor drive systems overcome the structural limitations of traditional internal combustion engine vehicles, offering benefits such as high energy efficiency, simplified body structure, and diverse drive modes [2]. Furthermore, as the drive units are distributed across each wheel, the vehicle’s weight distribution is more balanced, which helps to enhance handling stability and driving safety [3]. However, the unique mounting position of in-wheel motors also exposes them to more complex and demanding operating conditions during actual operation [4]. Due to direct exposure to road excitation and external environmental influences, in-wheel motors are subjected to multiple factors during operation, including frequent impact loads, vibration interference and temperature fluctuations [5]. As a common component in rotating machinery, bearings are inevitably prone to failure during operation [6]. Bearings are key components in in-wheel motors that provide support, transmission and load coupling: these operational conditions directly affect the reliability and safety of the entire vehicle system.

Consequently, research into the condition monitoring and fault diagnosis of in-wheel motor bearings holds significant engineering and practical value. In the field of mechanical fault diagnosis, vibration signals typically contain a wealth of information regarding the system’s state and fault characteristics. To achieve effective feature separation and frequency band analysis, researchers have proposed various signal decomposition methods. A multi-scale signal decomposition method based on the discrete wavelet transform was proposed, which performs hierarchical decomposition of vibration signals by constructing wavelet basis functions at different scales, thereby effectively characterising local features in the time–frequency domain [7]. Building upon the traditional wavelet analysis framework, the empirical wavelet transform has been developed [8,9] by constructing filter banks through adaptive spectral segmentation, thereby enhancing the method’s capability to handle non-stationary signals [10]. The empirical mode decomposition (EMD) method adaptively decomposes complex vibration signals into several intrinsic mode functions (IMFs) based on local signal features. This method has been widely applied in mechanical fault feature extraction [11,12]. Building on this, Ji proposed an ensemble empirical mode decomposition method, which enhances the stability of the mode decomposition results by introducing auxiliary noise and averaging multiple decompositions [13]. Dai further proposed a fully ensemble empirical mode decomposition method with adaptive noise, improving the noise addition strategy based on ensemble empirical mode decomposition to enhance the consistency of mode components and the accuracy of the decomposition [14]. Dragomiretskiy proposed the Variational Mode Decomposition (VMD) method, which constructs a signal decomposition model from the perspective of variational optimisation. By using an iterative solution, it obtains modal components with finite bandwidth characteristics and their centre frequencies, offering significant advantages in terms of theoretical framework and decomposition stability [15,16]. Furthermore, Nazari proposed Successive Variational Mode Decomposition (SVMD), which employs a mode-by-mode extraction strategy to sequentially separate modal components [17]. This method possesses a certain degree of adaptability in determining the number of modes and demonstrates good performance in terms of computational efficiency and robustness [18]. Although the aforementioned methods have achieved significant results in the field of non-stationary signal processing, in practical applications, the performance of SVMD remains highly dependent on the pre-set penalty factor. To address this limitation and overcome the constraints of manual parameter tuning, researchers have introduced various meta-heuristic optimisation algorithms to achieve automatic parameter optimisation. The Grey Wolf Optimizer (GWO) [19], the Sparrow Search Algorithm (SSA) [20], and the Crested Porcupine Optimizer (CPO) [21] have been widely applied. Among these, the CPO algorithm, as a relatively new method developed in recent years, offers advantages such as strong global search capabilities and high robustness [22]. By integrating multiple strategies, this algorithm enhances the quality of candidate parameter generation, providing a more efficient adaptive optimisation path for achieving the optimal parameter configuration of Successive Variational Mode Decomposition (SVMD). Consequently, current signal processing methods are difficult to apply to the field of fault feature extraction for in-wheel motor-driven electric vehicles; therefore, it is necessary to design a method capable of extracting fault features under complex conditions. To further evaluate the quality of the intrinsic modal functions (IMFs) following SVMD, this paper introduces an entropy-based evaluation criterion. Entropy, as a key tool for measuring signal complexity, is particularly suitable for the analysis of non-linear, non-stationary time series [23]. Its core advantage lies in its ability to reflect the uncertainty and irregularity of signals [24]. Depending on the method of complexity characterisation, entropy primarily includes Shannon entropy [25] and conditional entropy [26]. Among these, Shannon entropy is used to characterise the amount of information, whilst conditional entropy is used to describe the rate at which information is generated. Examples include dispersion entropy (DE) [27], Sample Entropy (SE) [28], and Rating Entropy (RE) [29]. Compared to traditional entropy metrics, RE performs better in characterising the dynamic characteristics of non-linear signals.

To address the aforementioned issues, this paper employs RE as an evaluation criterion to identify fault-sensitive IMF components from the SVMD results.

Deep learning is the most compelling research trend in the field of machine learning. Through deep architectures with multiple layers of non-linear data processing units, it can learn features from raw data. Thus, deep learning has become a promising tool for intelligent bearing fault diagnosis [30,31]. Deep learning models the complexity and internal correlations within datasets, thereby uncovering hidden information within the dataset to achieve classification or other objectives [32,33]. Currently, various types of deep learning methods exist, such as Long Short-Term Memory (LSTM) networks [34], Vision Transformers (ViTs) [35], Generative Adversarial Networks [36], and Deep Belief Networks [37], among others. The most representative of these is the Convolutional Neural Network (CNN) [38]. In traditional deep CNNs, as the number of layers increases, the model often faces the problem of vanishing gradients or exploding gradients, leading to severe degradation of the network [39]. To address this issue, He proposed the Residual Neural Network (ResNet) [40]. By introducing skip connections, the ResNet enables the neural network to adaptively degrade to a shallow network, whilst also significantly mitigating phenomena such as gradient explosion [41]. However, bearing fault diagnosis using a traditional ResNet is constrained by high parameter counts, high computational costs and low accuracy [42,43]. Zhang proposed a method based on a hybrid attention-enhanced residual network to diagnose faults in wind turbine gearboxes by highlighting fault features in the fundamental frequency bands of wavelet coefficients and convolutional channels. This effectively improved the non-linear feature extraction capability of deep convolutional networks [44]. Xie proposed an improved meta-ResNet with a sample-weighted bearing fault diagnosis method. They constructed a pooling layer using singular value decomposition to replace the traditional max-pooling layer in ResNet, thereby significantly enhancing the mapping relationship between feature maps and fault categories [45]. Moreover, to address cross-machine and cross-domain diagnostic challenges under complex unlabelled scenarios, advanced transfer learning approaches have shown great potential. Notable examples include the conditional distribution-guided adversarial transfer learning network [46] and spatial-channel collaborative multi-scale graph interaction deep transfer learning [47]. Furthermore, accurate fault diagnosis is an essential prerequisite for overall system safety. It provides a crucial foundation for advanced downstream strategies, such as the fixed-time adaptive fault-tolerant control of multi-mode systems [48].

Although ResNet demonstrates significant advantages, during the feature classification and decision-making stages, almost all existing models employ traditional multi-layer perceptrons or fully connected layers as classification heads [49,50]. The essence of fully connected layers lies in global linear weight mapping based on fixed activation nodes. When confronted with the highly coupled, extremely non-linear and fault feature boundaries generated by in-wheel motors in complex, high-noise environments, their ability to express non-linearity is severely limited. They are highly prone to overfitting under conditions with few samples, making it difficult to meet the high-precision diagnostic requirements of complex and variable scenarios [51].

Recently, the Kolmogorov–Arnold Network (KAN) has attracted significant attention due to its powerful non-linear fitting capability [52]. Unlike traditional neural networks, the KAN introduces learnable non-linear basis functions on network edges, thereby improving the representation of complex non-linear relationships. However, conventional KAN models based on B-spline functions still suffer from high computational complexity and low efficiency [53].

**Figure 1:**
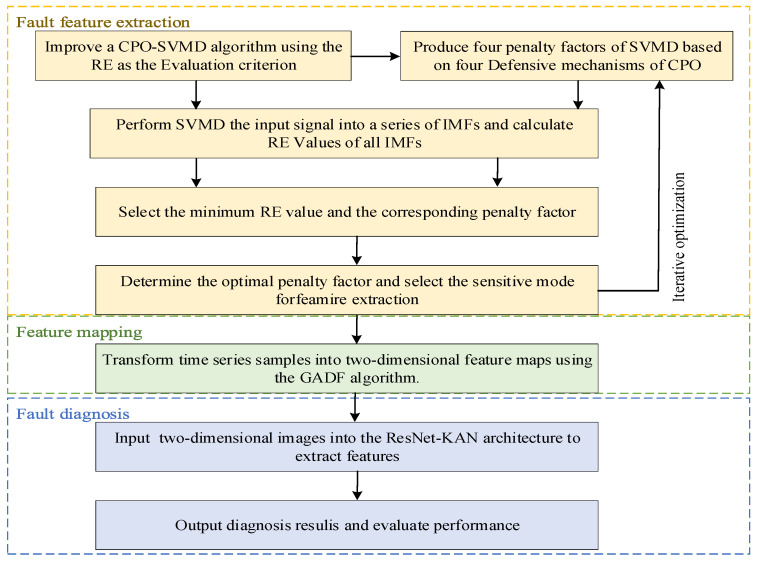
Framework of the fault diagnosis method for in-wheel motor bearings in electric vehicles based on improved SVMD and ResNet-KAN.

Therefore, to address the limitations of the traditional ResNet’s non-linear decision-making capabilities and the excessive computational overhead of the traditional KAN, this study proposes a novel approach to in-wheel motor bearing fault diagnosis based on the deep integration of two-dimensional feature maps derived from the Gramian angular difference field (GADF) [54] with ResNet-KAN, whose framework is shown in Figure 1. This paper aims to fully leverage the advantages of the ResNet in two-dimensional local feature extraction, whilst innovatively introducing a KAN based on bidirectional ReLU truncation optimisation to replace traditional fully connected layers. This significantly enhances the model’s non-linear expressive capability in the classification decision layer, thereby achieving high-precision and highly robust state identification of subtle bearing faults in complex and variable in-wheel motor operating scenarios.

The main contributions of this study are summarised as follows. First, a CPO-SVMD method is proposed to adaptively extract sensitive fault features while suppressing noise and modal aliasing. Second, a GADF-based mapping strategy is introduced to transform one-dimensional vibration signals into two-dimensional feature images while preserving temporal information. Finally, a lightweight ResNet-KAN model is constructed to achieve accurate and robust fault diagnosis under complex operating conditions.

## 2. Related Work

This chapter mainly introduces the theoretical foundations and implementation process of the proposed intelligent fault diagnosis framework for in-wheel motor bearings. Firstly, the SVMD method and RE are employed to achieve adaptive decomposition and sensitive feature extraction of vibration signals. Subsequently, STFT, WVD, S-transform, and GADF are introduced for time–frequency analysis and two-dimensional feature reconstruction of non-stationary signals. Finally, the deep feature extraction and intelligent classification mechanisms of ResNet, KAN, and the improved ReLU-KAN are presented, establishing the theoretical basis for the proposed fault diagnosis model.

### 2.1. Successive Variational Mode Decomposition

SVMD is a variant of the Variational Modal Decomposition (VMD) method, which employs a sequential decomposition strategy to iteratively extract modes. Each mode *u_L_* corresponds to an intrinsic modal function (IMF), obtained through the iterative extraction process in the Lth iteration, and occupies a unique frequency band. The original signal *x*(*t*) is represented as the sum of the extracted *L*-th mode *u_L_*(*t*) and the residual signal *x_r_*(*t*) [17], as shown below:

(1) $$x(t)=u_{L}(t)+x_{r}(t)$$

In SVMD, to ensure modal compactness, the bandwidth is minimised using the *L*_2_ norm of the square of the gradient of the demodulated signal, denoted as

(2) $$J_{1}={\left\|\partial_{t}\left[\left(\delta (t)+\frac{j}{\pi t}\right)*u_{L}(t)\right]e^{-j\omega_{L}t}\right\|}_{2}^{2}$$

where *ω_L_* is the centre frequency of *u_L_*(*t*), and * denotes the convolution operation. To prevent the residual signal *x_r_*(*t*) from retaining significant energy, a frequency-domain penalty *J*_2_ is introduced:

(3) $$J_{2}={\left\|\frac{1}{\alpha {\left(\omega -\omega_{L}\right)}^{2}}*x_{r}(t)\right\|}_{2}^{2}$$

Furthermore, to prevent the newly extracted mode from interfering with previously extracted modes, a separation constraint *J*_3_ is imposed:

(4) $$J_{3}=\sum_{i=1}^{L-1}{\left\|\frac{1}{\alpha {\left(\omega -\omega_{i}\right)}^{2}}*u_{L}(t)\right\|}_{2}^{2}$$

where *i* = 1, 2, …, *L* − 1. Consequently, the extraction of the *L*-th mode is formulated as the following minimisation problem subject to the constraints:

(5) $$\underset{u_{L},\omega_{L},x_{r}}{\min}\left\{\alpha J_{1}+J_{2}+J_{3}\right\}$$

(6) $$u_{L}(t)+x_{L}(t)=x(t)$$

By utilising Parseval’s theorem, this problem is solved using the Alternating Direction Method of Multipliers (ADMM). The analytical form for the update of the mode in the frequency domain is obtained from the following equation:

(7) $${\hat{u}}_{L}^{n+1}(\omega )=\frac{\hat{x}(\omega )+\alpha^{2}{\left(\omega -\omega_{L}^{n}\right)}^{4}{\hat{u}}_{L}(\omega )+\frac{1}{2}\hat{\lambda }(\omega )}{\left[1+\alpha^{2}{\left(\omega -\omega_{L}^{n}\right)}^{4}\right]\left[1+2\alpha {\left(\omega -\omega_{L}^{n}\right)}^{2}+\sum_{i=1}^{L-1}\alpha^{-2}{\left(\omega -\omega_{i}\right)}^{-4}\right]}$$

where ${\hat{u}}_{L}^{n+1}(\omega )$ is the Fourier transform of the updated mode $u_{L}^{n+1}(t)$, $\hat{x}(\omega )$ denotes the Fourier transform of the original signal *x*(*t*), and *λ*(*ω*) is the Fourier transform of the Lagrangian multiplier *λ*(*t*). The update formula for *ω_L_* is

(8) $$\omega_{L}^{n+1}=\frac{\int_{0}^{\infty }\omega {\left|u_{L}^{n+1}(\omega )\right|}^{2}d\omega }{\int_{0}^{\infty }{\left|u_{L}^{n+1}(\omega )\right|}^{2}d\omega }$$

The update formula for the Lagrangian multiplier is

(9) $${\hat{\lambda }}^{n+1}={\hat{\lambda }}^{n}+\theta \left[\hat{x}(\omega )-\left({\hat{x}}_{u}^{n+1}(\omega )+{\hat{u}}_{L}^{n+1}(\omega )+\sum_{i=1}^{L-1}{\hat{u}}_{i}^{n+1}(\omega )\right)\right]$$

where *θ* is the penalty parameter introduced by the ADMM, and ${\hat{x}}_{u}(\omega )$ denotes the unprocessed residual component; these iterative updates continue until convergence, thereby extracting the *L*-th eigenmode function.

### 2.2. Rating Entropy

In order to evaluate the sparsity and the impact of a signal simultaneously, the RE *f*(*u*_L_, *m*) is introduced [29], defined as

(10) $$f(u_{L},m)=\frac{1}{2}\left(\frac{H(B^{m})}{\ln(\frac{1}{2}m(m-1)+1)}+\frac{H(B^{m+1})}{\ln(\frac{1}{2}m(m+1)+1)}\right)$$

where *B^m^* denotes the reconstruction matrix formed by the modal *u_L_*; the detailed procedure for constructing the reconstruction matrix *B^m^* is described in the literature [25]; *H*(*B^m^*) refers to the entropy value; and *m* represents the embedding dimension, denoting the number of elements in each embedding vector. *m* is typically set to four.

### 2.3. Time–Frequency Analysis

Time–frequency analysis methods are tools used in signal processing to analyse features in the time–frequency domain, primarily for the characterisation of non-linear and non-stationary signals. Currently, the main time–frequency analysis methods include the short-time Fourier transform (STFT), the Wigner–Ville distribution (WVD) and the Stockwell Transform (S-transform).

The STFT is the cornerstone of time–frequency analysis, designed to overcome the inherent limitations of the traditional Fourier transform. The traditional Fourier transform provides only global frequency information and is unable to capture the time-varying frequency characteristics of non-stationary signals. The core idea of STFT is to use a sliding window to divide a non-stationary signal into several short-time approximately stationary segments, and then apply the Fourier transform to each segment to extract the frequency characteristics at a specific point in time. The calculation formula [55] is as follows:

(11) $$\mathrm{STFT}(t,\omega )=\int_{-\infty }^{+\infty }x(\tau )w(\tau -t)e^{-j\omega t}d\tau$$

where STFT(*t*, *ω*) denotes the corresponding amplitude at time *t* and frequency *ω*, *τ* is the integration variable representing the time axis, *w*(*τ* − *t*) is the window function, and *x*(*τ*) is the signal to be analysed. However, the STFT is constrained in its choice between temporal and frequency resolution; it is impossible to achieve optimal performance in both dimensions simultaneously. Furthermore, as the length of the window function is fixed, it cannot adaptively adjust the window width when analysing non-stationary signals, resulting in suboptimal performance [56].

Unlike the STFT, the WVD is a quadratic time–frequency distribution that is not affected by the window function and can provide high-resolution time–frequency information. The specific calculation method [57] is as follows:

(12) $$WVD_{x}(t,\omega )=\frac{1}{2\pi }\int_{-\infty }^{+\infty }x(t+\frac{\tau }{2})x^{*}(t-\frac{\tau }{2})e^{-j\omega t}dt$$

Here, *x*(·) and *x*^∗^(·) represent the input signal and its complex conjugate, respectively. It can be seen from Equation (11) that the WVD determines the energy distribution of the signal in the time–frequency domain by analysing the correlation of the signal at different time points. As the WVD is not constrained by a window function, it is capable of achieving high temporal and frequency resolutions; however, this also leads to the problem of cross-term interference during time–frequency analysis, particularly when analysing vibration signals with a low signal-to-noise ratio.

The S-transform, as an extension and development of the short-time Fourier transform, employs a Gaussian window function with a window width proportional to the inverse of the frequency, thereby overcoming the limitation of the fixed window width in the short-time Fourier transform. The specific calculation method [58] is as follows:

(13) $$S(t,\omega )=\int_{-\infty }^{+\infty }x(\tau )\frac{\left|\omega \right|}{2\pi \sqrt{2\pi }}e^{-\frac{{\left(\tau -t\right)}^{2}\omega^{2}}{8\pi^{2}}e^{-j\omega t}d\tau }$$

where *τ* is time, *j* is the imaginary unit, and *t* is the centre position of the Gaussian window function.

The Gramian angular field (GAF) is a method that combines coordinate transformation with the Gramian matrix, enabling the conversion of one-dimensional time data into a two-dimensional plot. This technique is capable of extracting features that reflect dynamic changes in both the time and frequency domains. The GAF preserves the temporal dependency of the signal while effectively capturing feature information from one-dimensional data. The GAF is divided into two types, GADF and GASF (Gramian angular summation field, GASF), with GADF demonstrating superior classification capabilities compared to GASF. This study employs the GADF image encoding method for image transformation, with the specific formula [59] as follows.

The values in the one-dimensional data sequence *X* are scaled to the intervals [−1, 1] and [0, 1] using specified expressions:

(14) $${\tilde{x}}^{i}=\frac{x_{i}-\max(X)+x_{i}-\min(X)}{\max(X)-\min(X)}$$

where *x_i_* is the original vibration signal at time *t*, ${\tilde{x}}_{i}$ denotes the value normalised according to Equation (14), and represents the signal at that time after scaling.

After converting this series of normalised data to polar coordinates, the time series *X* can be expressed in polar coordinates.

(15) $$\left\{\begin{array}{c} \varphi_{i}=\arccos({\tilde{x}}^{i}),-1\leq {\tilde{x}}^{i}\leq 1,{\tilde{x}}^{i}\in \tilde{X}\\ r_{i}=\frac{t_{i}}{N},t_{i}\in N \end{array}\right.$$

where *φ_i_* denotes the angle in polar coordinates mapped from *x_i_*, *t_i_* denotes the timestamp code for that point, used as a factor to adjust the radial range in the polar coordinate system, *N* is a constant factor, and *r_i_* is the radius mapped from the timestamp *t_i_*. By utilising the phase difference relationship described in Equation (15) to identify temporal correlations between different time points, the target image is ultimately generated.

(16) $$\mathrm{GADF}=\left[\begin{array}{cccc} \sin(\varphi_{1}-\varphi_{1}) & \sin(\varphi_{1}-\varphi_{2}) & \cdots & \sin(\varphi_{1}-\varphi_{n})\\ \sin(\varphi_{2}-\varphi_{1}) & \sin(\varphi_{2}-\varphi_{2}) & \cdots & \sin(\varphi_{2}-\varphi_{n})\\ \vdots & \vdots & \ddots & \vdots \\ \sin(\varphi_{n}-\varphi_{1}) & \sin(\varphi_{n}-\varphi_{2}) & \cdots & \sin(\varphi_{n}-\varphi_{n}) \end{array}\right]$$

It can be seen that this encoding method preserves the temporal order of the two-dimensional image, from top-left to bottom-right. The original information is retained at the positions along the main diagonal, whilst the other regions express the relationships between different time sequences. For a vibration signal with an original time series length of *n*, GADF encoding yields a matrix of dimensions *n* × *n* [60].

### 2.4. ResNet

The core idea of the ResNet is to introduce identity mappings, so that the network no longer learns the target mapping function of the underlying layer directly, but instead learns the residual mapping.

We suppose that the input to a certain layer of the network is *x*, and the low-level mapping feature to be learned is *H*(*x*). In traditional convolutional networks, this layer is required to directly fit *H*(*x*). In contrast, in a residual module, the network is designed to fit the residual function *F*(*x*) [40], defined as shown in Equation (17):

(17) $$F\left(x\right)=H\left(x\right)-x$$

Consequently, the originally desired mapping can be obtained by element-wise addition of the learned residual features with the input features; that is, the expression for the final output y of the residual block is

(18) $$y=F\left(x,\left\{W_{i}\right\}\right)+x$$

where *F*(*x*,*W_i_*) is the non-linear mapping along the residual path, comprising convolution, batch normalisation and the ReLU activation function, and *x* is the identity mapping propagated across layers.

### 2.5. Kolmogorov–Arnold Networks

The Kolmogorov–Arnold representation theorem states that any continuous multivariate function defined on a bounded closed interval can be represented as a superposition of a finite number of continuous univariate functions. Let *f*(*x*_1_, *x*_2_, …, *x_n_*) be a continuous function defined on a bounded *n*-dimensional region, where *x*_1_, *x*_2_, …, *x_n_* denote the *n* input variables of the function, and *n* is the dimension of the input space. According to this theorem, the function *f*(·) [61] can be expressed as

(19) $$f(x_{1},\cdots ,x_{n})=\sum_{q=1}^{2n+1}\phi_{q}(\sum_{p=1}^{n}\phi_{q,p}(x_{p}))$$

**Figure 2:**
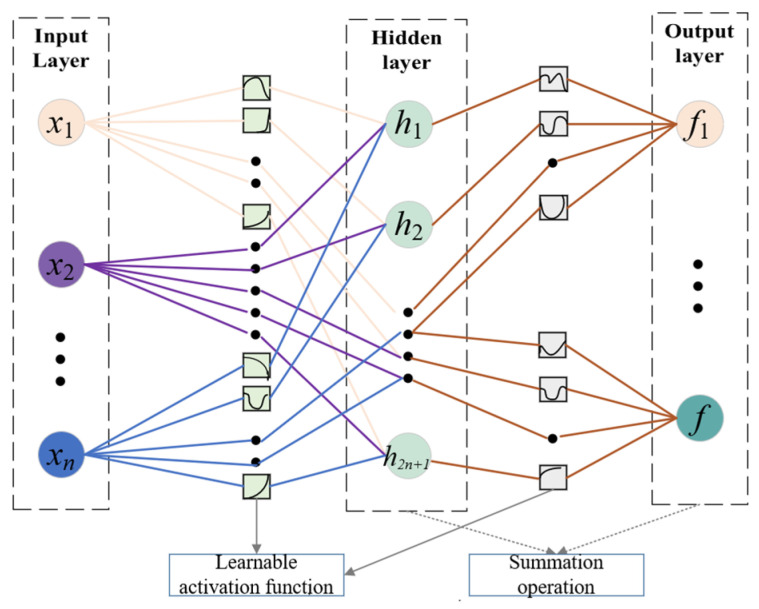
Schematic diagram of KAN architecture.

In particular, the outer summation index q ranges from 1 ≤ *q* ≤ 2*n* + 1, where 2*n* + 1 is the upper bound on the number of functions required to theoretically guarantee the approximation capability; ϕ*q*(·) denotes the *q*-th univariate continuous function, which is used to perform a non-linear mapping on the results of the inner summation; the inner summation indices *p* range from 1 to *n*, corresponding to each input variable; and ϕ*q*, *p*(*x_p_*) denotes the univariate continuous function constructed for the *p*-th input variable in the group, where the input is the single variable *x_p_* and the output is a scalar. This expression demonstrates that a complex multidimensional function *f*(·) can be precisely approximated through the linear superposition and nested mapping of several one-dimensional functions, thereby transforming high-dimensional modelling problems into low-dimensional function composition problems. In the KAN, the fixed activation functions on nodes in traditional neural networks are removed and replaced by learnable one-dimensional functions on the network’s connecting edges, thereby providing a solid mathematical foundation for constructing non-linear neural networks with more compact structures and greater expressive power. The topology of the KAN is shown in Figure 2.

In traditional KAN constructions, B-spline basis functions are typically employed to realise a parametric representation of the one-dimensional learnable function *ϕ*(·). Let the input variable be x∈ℝ; then, the one-dimensional non-linear mapping function can be expressed as

(20) $$\phi (x)=\sum_{i=1}^{M}c_{i}B_{i}(x)$$

where *M* denotes the number of spline basis functions; *B_i_*(*x*) is the *i*-th B-spline basis function, which is a piecewise polynomial function with local support; and the trainable coefficient parameters correspond to the basis function. By adjusting the coefficients *c_i_*, the shape of the function *ϕ*(*x*) can be flexibly altered to approximate any continuous function.

However, in practical applications for industrial bearing fault diagnosis, traditional B-spline-based KANs have revealed significant shortcomings. Firstly, the recursive computation process of B-splines is extremely complex, resulting in enormous computational overhead during both forward inference and backpropagation. Secondly, spline interpolation relies on a complex dynamic mesh updating mechanism, making it difficult to utilise the highly optimised matrix operations in GPUs for parallel acceleration, which greatly limits its deployment efficiency in deep feature extraction models.

### 2.6. KAN Based on Bidirectional ReLU

To address the computational efficiency and parallelisation bottlenecks of traditional KANs whilst retaining their non-linear boundary-fitting capabilities, this paper proposes an improved ReLU-KAN architecture in the decision-making and classification head of the model. This architecture utilises a combination of piecewise linear activation functions to construct local smoothing basis functions. Specifically, for a given local grid interval [*s*, *e*], where s is the lower boundary of the grid and *e* is the upper boundary, this paper constructs a strictly bounded local basis function by multiplying two ReLU truncation functions with opposite directions:

(21) $$\phi_{bump}(x)=\left(\mathrm{ReLu}(e-x)\cdot \mathrm{ReLu}{\left(x-s\right)}^{2}\right)\cdot r$$

In this context, ReLU(·) denotes the rectified linear unit function; r is a normalisation scale factor dependent on the grid step size *h*, defined as *r* = (2/*h*)^4^. This design is mathematically equivalent to the local support property of B-splines, but is extremely simple to compute. When implementing efficient dimensional mapping for one-dimensional features, the ReLU-KAN does not employ inefficient iterative traversal; instead, it reshapes the feature inputs along the grid dimension and utilises two-dimensional equivalent convolution in place of fully connected weight matrices. This enables parallel weighted summation of local basis function activation values, significantly enhancing hardware computational efficiency.

Furthermore, to balance the global linear propagation of features with local non-linear fine-tuning, this paper introduces a dual-branch architecture within the ReLU-KAN layer. The final output feature *Y* is expressed as

(22) $$Y=\lambda_{s}\cdot Conv2D(\phi_{bump}(X))+\lambda_{p}\cdot \mathrm{SiLU}({XW}_{p})$$

where *φ_bump_*_(·)_ is the feature vector input to the backbone network; *W_p_* is the weight matrix of the linear branch; SiLU(·) is the Sigmoid activation function, expressed as SiLU(*x*) = *x*·(*x*); and *λ_s_* and *λ_p_* are the learnable scaling coefficients for the two branches, respectively.

Through the above improvements, the ReLU-KAN proposed in this paper not only avoids the computational overload caused by traditional spline interpolation but also, by virtue of its edge-based activation mechanism, demonstrates non-linear decision-making capabilities far surpassing those of traditional fully connected layers when processing high-dimensional features in bearing vibration signals where periodic and impact components are highly coupled.

## 3. Validation and Comparative Analysis of the CPO-SVMD Method Based on Simulation Signals

This chapter mainly validates the effectiveness and superiority of the proposed CPO-SVMD method using simulated bearing fault signals under strong noise interference. Firstly, a simulated inner-race fault signal is constructed to reproduce the severe noise environment of in-wheel motors. Subsequently, different optimisation algorithms are introduced into the SVMD framework for comparative analysis, and the sensitive modes extracted by each method are evaluated using envelope spectra, RE, SNR, and kurtosis indicators. Finally, the influence of the extracted sensitive signals on subsequent GADF image construction and deep feature learning is analysed.

To validate the effectiveness of CPO-SVMD, a simulated bearing signal with an inner ring fault was constructed, as shown below:

(23) $$\left\{\begin{array}{l} x(t)=s(t)+n(t)=\sum_{i=1}^{N}A_{i}h(t-iT)+n(t)\\ A_{i}=1+A_{0}\cos(2\pi f_{r}t)\\ n(t)=e^{\left(-Ct\right)}\cos(2\pi f_{n}t) \end{array}\right.$$

where *s*(*t*) is the impact signal and *n*(*t*) is the noise signal. The amplitude of the fault characteristic impact signal *A*_0_ is 0.3, the resonance frequency *f_n_* is 4 kHz, the rotational frequency *f_r_* is 30 Hz, the inner-race fault frequency *f_i_* is 120 Hz, and *C* is 700. An SNR of −14 dB was selected to simulate the severe noise environment characteristic of in-wheel motors, as the motor is subject to direct road excitation and structural resonance. Furthermore, the sampling frequency *f_s_* is 16 kHz, and the number of sampling points is 8192.

**Figure 3:**
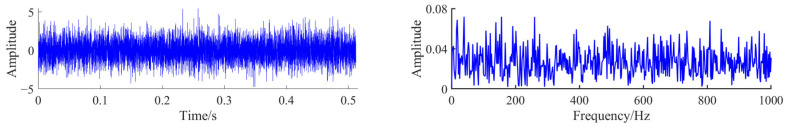
A simulation-bearing signal with an inner-race fault: time waveform of *x*(*t*); envelope spectrum of *x*(*t*) within frequency range of 0 to 1000 Hz.

Figure 3 presents the time-domain waveform and the corresponding envelope spectrum of the simulated inner-race fault signal. Due to the strong background noise under the −14 dB SNR condition, the periodic fault impulses are difficult to identify directly from the time-domain waveform. In the frequency domain, although the inner-race fault characteristic frequency near 120 Hz and its harmonic components can be partially observed, they are significantly masked by broadband noise interference. The maximum number of iterations was set to 20. However, the optimisation process terminated early once the RE reduction rate satisfied the predefined convergence criterion. During each iteration, the penalty factor corresponding to the minimum RE value was selected as the current optimal solution and used for the subsequent iteration. The optimal penalty factors obtained using the four optimisation algorithms are summarised in Table 1. For comparison, within the constructed CPO-SVMD optimisation framework, SSA, GWO, PSO and CPO were employed, respectively, to select the parameters of SVMD, thereby constructing the comparative models, SSA-SVMD, GWO-SVMD, PSO-SVMD and CPO-SVMD. To ensure the fairness of the comparative experiments, all algorithms were executed under the same SVMD parameter settings: the delay parameter was set to 0, the convergence threshold to 1 × 10^−6^, and the number of consecutive unchanging modes at convergence to four.

**Figure 4:**
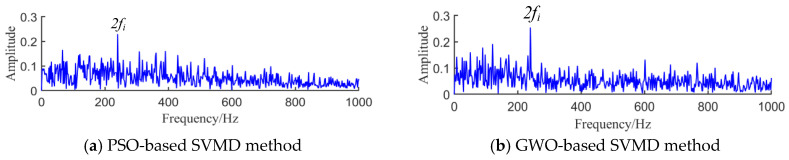

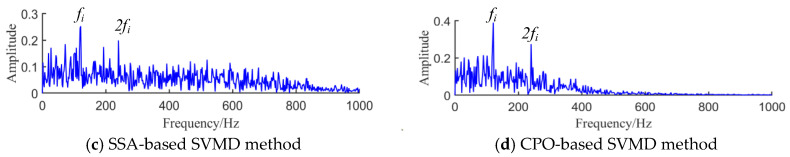
ISF values of different IMFs with the identified sensitive mode (**left**) and the corresponding envelope spectrum showing fault feature frequency (**right**).

**Figure 5:**
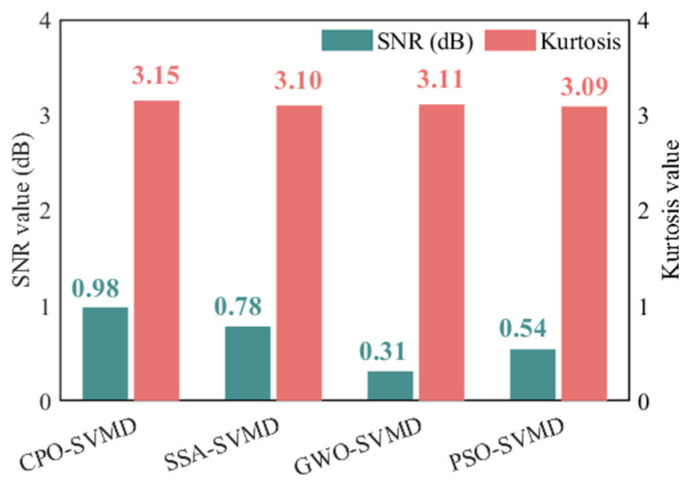
SNR and kurtosis metrics of the sensitive mode obtained by four methods.

Upon convergence, the modes possessing minimal *f*(*u*_L_, *m*) are regarded as sensitive modes. The envelope spectra of these sensitive modes, along with the *f*(*u*_L_, *m*) values for each mode following convergence using different methods, are shown in Figure 4. It is worth noting that the number of IMFs varies across different decomposition results, ranging from as many as 35 to approximately 30. This variation is due to the fact that the optimal penalty factor determined by different methods directly influences the decomposition depth of SVMD. Furthermore, quantitative metrics such as the SNR and kurtosis are used to evaluate the signals processed by various methods, as shown in Figure 5. Among these, the signal-to-noise ratio (SNR) and kurtosis of the sensitive modes extracted using the CPO-SVMD method were relatively superior, with the SNR increasing by at least 1.3% and the kurtosis improving by at least 25.6%. The SSA-SVMD method performed second best, whilst the GWO-SVMD method yielded the poorest results. This demonstrates that the proposed CPO-SVMD method possesses outstanding capabilities in extracting fault-related features.

CPO-SVMD suppresses background noise while preserving periodic fault impulses by adaptively matching the optimal bandwidth of transient fault features. The resulting improvements in kurtosis and signal-to-noise ratio indicate that the extracted signals contain clearer fault-related information. When transformed into GADF images, these purified signals generate more structured and distinguishable texture patterns, thereby enhancing inter-class separability in the two-dimensional feature space and reducing the influence of noise interference on deep feature learning.

## 4. Performance Evaluation and Experimental Verification of the Proposed Model

This chapter mainly introduces the experimental validation and intelligent fault diagnosis framework for in-wheel motor bearings under real operating conditions. Firstly, an in-wheel motor experimental bench and fault simulation scheme are established to collect vibration signals under different fault states and operating conditions. Subsequently, the STFT, WVD, S-transform, and GADF are employed to construct and compare two-dimensional feature maps, while SSIM is introduced to quantitatively evaluate the discriminative capability of different time–frequency representations. Finally, a ResNet-KAN-based intelligent fault diagnosis model is proposed, and its effectiveness, robustness, and generalisation abilities are comprehensively verified through model comparison experiments, t-SNE visualisation, cross-condition validation, and ablation studies.

### 4.1. Experimental Bench for In-Wheel Motors

**Figure 6:**
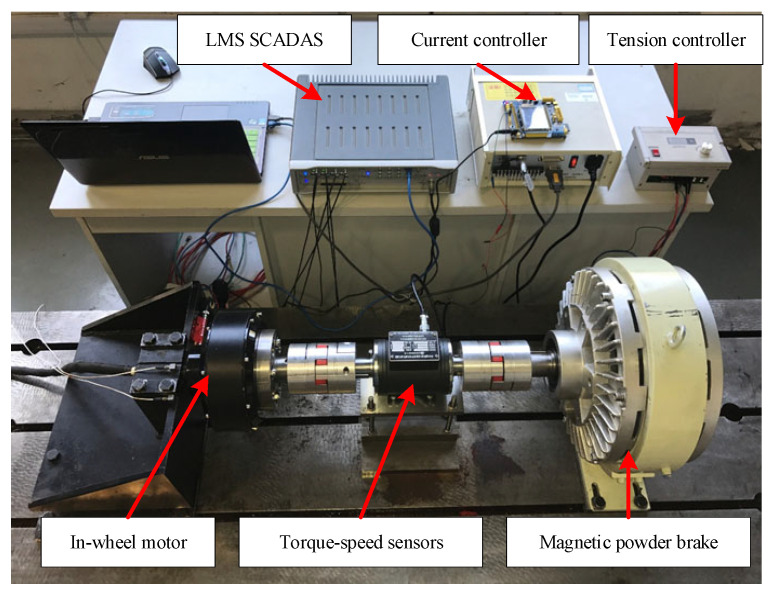
In-wheel motor experimental test bench.

Figure 6 shows an experimental test bench for in-wheel motor fault diagnosis. It consists primarily of an in-wheel motor and an STM32 microcontroller, powered by a set of power batteries. Unlike conventional centrally driven motors, in-wheel motors adopt an integrated outer-rotor structure in which the motor is directly mounted inside the wheel hub. Consequently, the supporting bearings are subjected not only to electromagnetic torque, but also to complex road-induced excitations, variable radial and axial loads, and continuous vibration impacts during vehicle operation. In addition, the in-wheel motor bearings typically operate under variable-speed and variable-load conditions, especially in low-speed and high-torque driving scenarios, which significantly increase the difficulty of extracting weak fault features from vibration signals. Furthermore, an accelerometer (model: PCB333B30; sensitivity: 100 mV/g) was mounted vertically on the bracket securing the in-wheel motor stator shaft, and a torque–speed sensor was connected to measure the actual torque and rotational speed. To quantitatively characterise the fault states of the in-wheel motors, a single-point defect (width 0.3 mm, depth 0.15 mm) was created sequentially on the inner ring, rolling elements and outer ring of three bearings specifically designed for in-wheel motors (model: DU2505237), after which they were installed on the stator shafts of each in-wheel motor by qualified personnel. The defect dimensions of 0.3 mm in width and 0.15 mm in depth were specifically selected to simulate the early weak fault stage commonly encountered in practical engineering applications, thereby enabling the evaluation of the proposed method under weak fault feature conditions. During the experiment, these defective bearings were sequentially installed in otherwise normal in-wheel motors to simulate different fault conditions. The motor speed was controlled via an STM32 microcontroller, operating at speeds of 100, 200, …, 700 r/min (or thereabouts). In this experiment, a 16-channel LMS multifunctional data acquisition system was employed, which synchronously acquired vibration signals via a data acquisition terminal. The sampling frequency was 100 kHz, with a sampling duration of 20 s.

In the World Light Vehicle Test Procedure [62], the maximum speed under urban conditions is 56.05 km/h. The corresponding rotational speed of the in-wheel motor is calculated using the following formula:

(24) $$\omega_{r}=\frac{1000}{2\pi R\times 60}v$$

where *R* represents the tyre radius in metres, and *v* represents the vehicle speed in kilometres per hour. Taking a 225/55/R18 tyre as an example, its effective radius is approximately 0.35 metres. Substituting these values into the formula yields a motor speed of approximately 500 r/min. At this motor speed, the rotational frequency is 8.3 Hz. Calculations show that the corresponding theoretical natural frequencies for the bearings are: 55.8 Hz for inner ring failure, 33.4 Hz for outer ring failure, and 41.1 Hz for rolling element fault.

### 4.2. Construction of a Two-Dimensional Feature Map of In-Wheel Motor Bearing Vibration Signals

Following the preprocessing of bearing fault signals in real operating environments using the CPO-SVMD extraction method, one-dimensional vibration signals are mapped into a two-dimensional space by introducing the GADF. Based on experimental data from static and dynamic operating conditions, the effectiveness of feature construction using different methods is analysed and compared, and the validity of the two-dimensional map constructed using the GADF is quantitatively verified using the structural similarity index.

To validate the advantages of the two-dimensional data reconstruction method in the time–frequency domain proposed in this study, two-dimensional images were constructed using STFT, S-transform, WVD, and GADF, and a comparison was carried out. Vibration signal data from an in-wheel motor bearing operating under static conditions at a medium speed of 500 r/min were utilised, as this condition contains clear periodic rotational components and fault-induced impact characteristics.

A sample size of 12,000 points was selected, primarily to ensure coverage of the periodic features. For the bearing vibration signal at 500 r/min, the rotational frequency is 8.33 Hz, corresponding to a period of 0.12 s. Selecting 12,000 points as the sample size covers one full rotational cycle, enabling the effective capture of periodic features related to the rotational frequency within the time–frequency matrix.

First, the parameters for each method were set based on the characteristics of the experimental data: the STFT window function was set to the Hamming window, with a window length of 512, an overlap of 256, and a single-pass Fourier transform sample size of 1024; the time and frequency resolutions of the Wigner–Ville distribution were both determined by the sampling frequency and signal length; subsequently, two-dimensional plots of the time–frequency matrices for each method were generated, as visualised below.

**Figure 7:**
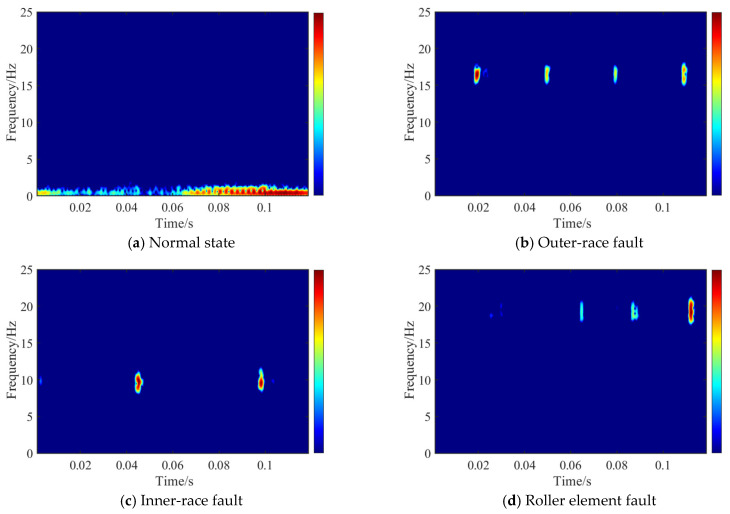
Time–frequency spectrograms based on STFT.

Figure 7 shows the time–frequency distribution results obtained using the STFT. Overall, under static operating conditions, the signal spectrum structure is relatively stable, and the energy distribution exhibits strong continuity. Under normal conditions, energy is primarily concentrated in the low-frequency region and is uniformly distributed along the time axis, with no obvious abrupt changes, reflecting the smooth operation of the system. In contrast, under outer-race, inner-race and rolling element fault conditions, intermittently enhanced energy bands appear in the time–frequency spectrogram; these bands are discretely distributed along the time axis, corresponding to local impact responses caused by fault excitation.

**Figure 8:**
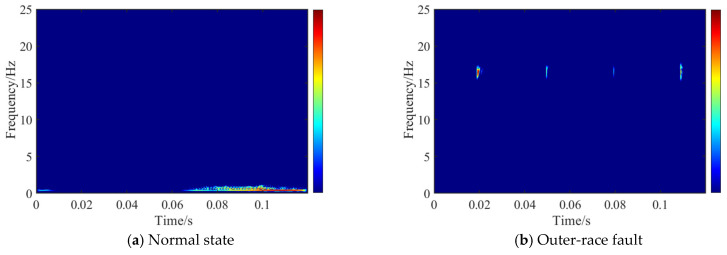

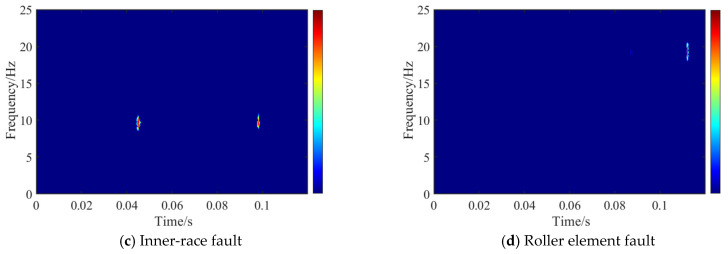
Time–frequency spectrograms of four states of the in-wheel motor based on WVD.

Figure 8 shows the time–frequency plots corresponding to four different states of an in-wheel motor based on the WVD. As the WVD possesses a high degree of time–frequency resolution, its energy distribution is more concentrated and local details are more pronounced. Under normal conditions, the energy manifests as a highly concentrated single dominant structure, with a relatively clear time–frequency distribution; however, under fault conditions, localised energy enhancement can be observed. However, at the same time, the figure reveals distinct cross-interference components, which are particularly pronounced in the case of inner-ring faults; these interference components manifest as non-physical oscillatory structures in the time–frequency domain. Consequently, although the WVD enhances resolution, the introduction of these cross-terms obscures the true fault characteristics to some extent, hindering subsequent identification.

**Figure 9:**
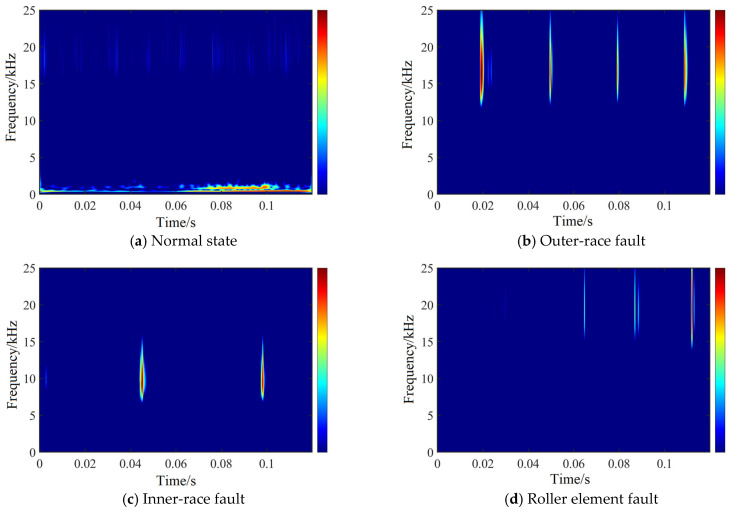
Time–frequency spectrograms of four states of the in-wheel motor based on the S-transform.

Figure 9 shows the time–frequency spectrograms corresponding to the four states of the in-wheel motor based on the S-transform. In the time–frequency spectrograms of the four states, the continuous bright band at the bottom in the normal state primarily corresponds to the low-frequency fundamental vibration component during bearing operation, whereas the bright vertical bands appearing in the time–frequency spectrograms for outer-ring and inner-race faults are transient impact signals caused by bearing operation or friction due to local damage. However, a comparison of Figure 8a–d reveals that, although the position and brightness of the transient-impact bright bands differ slightly across different fault states, the overall texture profiles and energy distribution structures of the four images are very similar.

**Figure 10:**
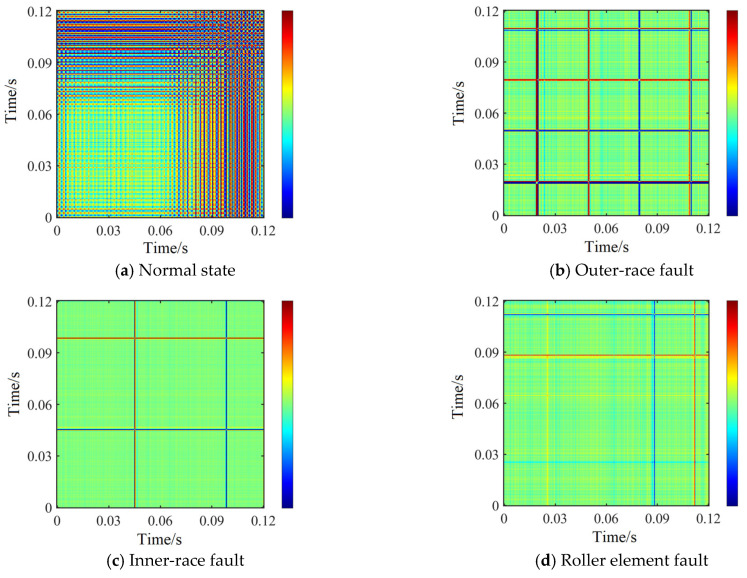
2D feature maps of four states of the in-wheel motor based on GADF.

Figure 10 shows the 2D feature maps of the four states of the in-wheel motor based on the GADF. Under normal conditions, the image exhibits a dense and chaotic grid-like texture, whereas under outer-ring, inner-ring and rolling element fault conditions, regular structural patterns gradually emerge in the image, and there are distinct differences in the distribution of textures corresponding to different failure types.

To intuitively demonstrate the discriminative power of the temporal matrix grayscale images under different states, the structural similarity index (SSIM) was employed to perform pairwise comparisons of the temporal matrix grayscale images across different states, with quantitative analysis used to evaluate the relative merits of each method. The calculation method [63] is as follows:

(25) $$\mathrm{SSIM}(a,b)=\frac{\left(2\mu_{a}\mu_{b}+C_{1})(2\sigma_{ab}+C_{2}\right)}{\left(\mu_{a}^{2}+\mu_{b}^{2}+C_{1})(\sigma_{a}^{2}+\sigma_{b}^{2}+C_{2}\right)}$$

In this context, time–frequency plots a and b are the objects for which the SSIM is calculated; *μ_a_* and *μ_b_* are the means of *a* and *b*, respectively; *σ_a_* and *σ_b_* are the standard deviations of *a* and *b*, respectively; *σ_ab_* is the covariance between *a* and *b*; and *C*_1_ and *C*_2_ are constants used to avoid situations where the denominator becomes zero. The SSIM takes values between −1 and 1. When the SSIM value is close to one, it indicates that the information in the two plots is similar, when the SSIM value is −1, it indicates that the information in the two plots is completely opposite [64]. Based on this, the SSIM values for the images under different methods are as follows.

As can be seen from Table 2, the SSIM values between the two-dimensional images generated using the STFT, WVD and S-transform are generally high, with the STFT reaching as high as 0.96 or above. In contrast, the GADF feature map exhibits extremely significant differences; the SSIM values between the normal state and outer-race faults or rolling element faults even show a negative correlation, being far lower than the SSIM values obtained using the other three time–frequency analysis methods. Furthermore, when distinguishing among different fault types, the SSIM values of the STFT, WVD, and S-transform remain within the range of 0.68–0.96, whereas those of the GADF are consistently confined to a much lower range of 0.05–0.11. In particular, the SSIM value between outer-race faults and rolling element faults is only 0.0559, demonstrating the strong discriminative capability of the GADF. Moreover, the mean SSIM value of the GADF is only 0.0227, which is substantially lower than those of the STFT, WVD, and S-transform. These results demonstrate that the GADF-based feature maps possess superior inter-class discrimination capabilities and are more suitable for intelligent fault diagnosis tasks.

### 4.3. A ResNet-KAN Method for In-Wheel Motor Bearing Fault Diagnosis

The bearing vibration signals acquired from the in-wheel motor drive system are converted into two-dimensional feature maps using the Gramian angular difference field (GADF) method, which are then used as the inputs of the intelligent fault diagnosis model. Based on this framework, a ResNet-KAN fault diagnosis model is proposed. The proposed model employs a lightweight ResNet as the backbone network for feature extraction and adopts a hybrid pooling strategy to replace conventional global average pooling. In the classification stage, an improved ReLU-KAN architecture based on the Kolmogorov–Arnold representation theorem is introduced as the non-linear decision head. By combining ReLU-based basis functions with a residual bypass structure, the proposed model achieves an enhanced non-linear representation capability while maintaining computational efficiency.

To validate the effectiveness of the proposed method, experiments including model comparison under identical operating conditions, cross-condition validation, t-SNE visualisation, and ablation studies were conducted.

**Figure 11:**
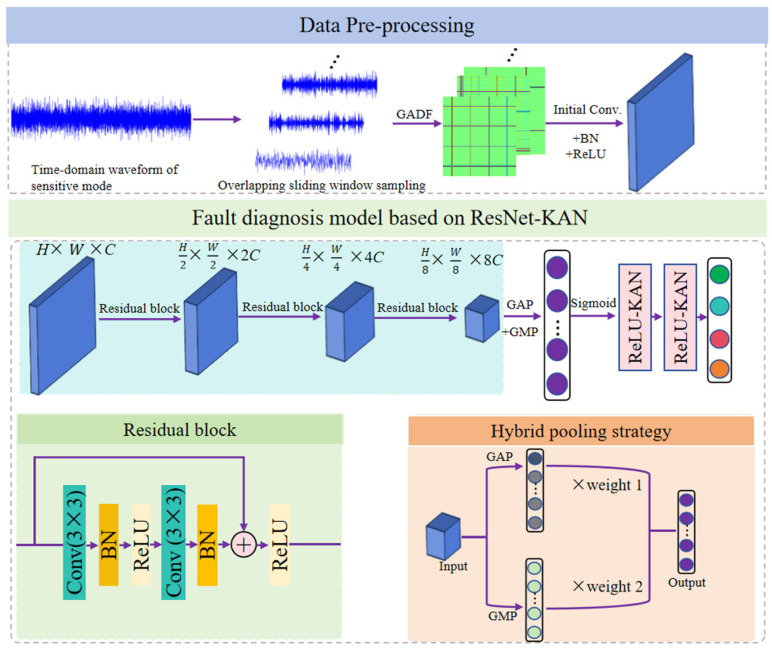
Overall architecture of the ResNet-KAN-based fault diagnosis model.

Firstly, the vibration signals collected from the in-wheel motor under complex noisy conditions are processed using the GADF to generate two-dimensional feature maps. The generated GADF images preserve the temporal dependency of the original signals while effectively highlighting periodic modulation and transient fault impact characteristics, thereby providing suitable inputs for subsequent deep convolutional networks. As shown in Figure 11, the proposed ResNet-KAN model consists of an initial convolutional layer, four residual layers containing eight residual blocks, a hybrid pooling layer, and two ReLU-KAN layers. The front-end network employs a 3 × 3 convolution, batch normalisation, and a ReLU activation function to extract shallow features without changing the input resolution. The extracted features are subsequently propagated through four residual layers. Each residual block contains two convolutional layers and an identity shortcut connection, which effectively alleviates the gradient vanishing problem in deep networks [65]. As features are propagated through the four residual layers, the network employs downsampling operations to halve the size of the feature maps at each stage, whilst doubling the number of channels, ultimately outputting high-level abstract spatial features of dimensions 32 × 28 × 28. To enhance deep feature aggregation while controlling model complexity, a hybrid pooling strategy combining global average pooling (GAP) and global max pooling (GMP) is adopted. GAP preserves global structural information but may weaken transient fault impulses, whereas GMP is more sensitive to local impulsive features but may lose steady-state information. Therefore, the features extracted by GAP and GMP are fused with equal weighting and compressed into a 1 × 1 × 32 tensor, which is then flattened into a 32-dimensional feature vector. A Sigmoid mapping layer is further introduced to constrain the feature values into a bounded interval, ensuring compatibility with the activation grid of the subsequent KAN layers. The main innovation of the proposed model lies in the classification stage, where traditional fully connected layers are replaced with a two-layer ReLU-KAN decision head. Each ReLU-KAN layer adopts a dual-branch architecture.

Non-linear basis function branch: By multiplying two ReLU functions with opposite directions, a strictly bounded local basis function is constructed, which is subsequently multiplied by the KAN scaling factor. This branch replaces the traditional B-spline function, retaining powerful high-dimensional non-linear fitting capabilities whilst significantly improving computational speed.

Linear residual compensation branch: The input features are multiplied by a linear matrix, activated by the SiLU function, and then multiplied by a residual scaling factor. This branch ensures the stable transmission of fundamental linear features within the network. The two feature streams are ultimately summed element-wise and output. Following this high-order feature space transformation by the decision head, the model ultimately outputs prediction results for four nodes, corresponding to four states of the in-wheel motor bearings (see Table 3).

### 4.4. Experimental Validation and Performance Analysis

To evaluate the diagnostic model’s diagnostic capability, stability, generalisation ability and superiority, validation under identical operating conditions, t-SNE visualisation, cross-validation and ablation experiments were conducted in sequence.

#### 4.4.1. Model Comparison and Validation Under Identical Operating Conditions

**Figure 12:**
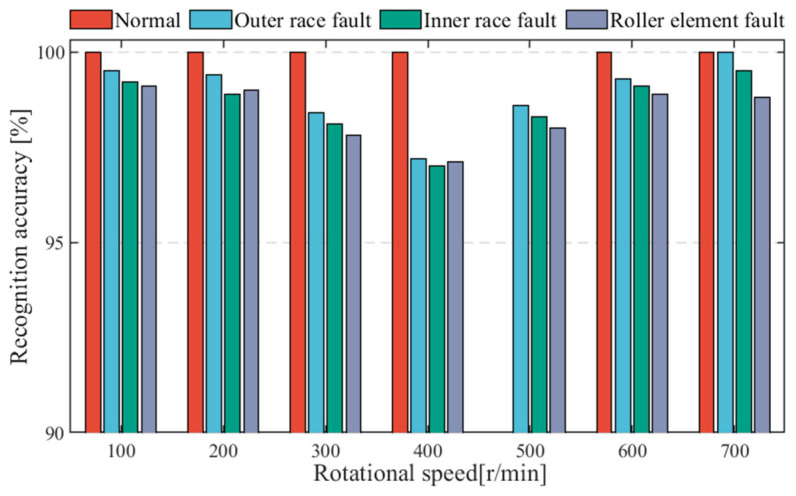
Recognition accuracy of the four states of the in-wheel motor under the same operating conditions.

Sliding-window segmentation was applied to the raw one-dimensional vibration signals collected under four bearing conditions. For each 20 s vibration signal, 12,000 sampling points were selected per sample, yielding 331 samples for each condition after segmentation. To satisfy the input requirements of the two-dimensional CNN, all one-dimensional samples were transformed into GADF images. Firstly, for each operating condition, the generated images were randomly shuffled and divided into training, testing, and validation sets with a ratio of 7:1.5:1.5, corresponding to 231 training samples, 50 testing samples, and 50 validation samples for each bearing state. The hyperparameters for the ResNet-KAN model were configured as follows: the Adam optimiser was selected, with 100 iterations, a batch size of 32, an initial learning rate of 0.001, and a weight decay coefficient of 1 × 10^−4^ to implement *L*^2^ regularisation. Furthermore, the hardware configuration for the fault diagnosis model in this study is as follows: the operating system is Windows 10, the GPU is an RTX 1650, equipped with 16 GB of system memory, and the deep learning framework used is PyTorch 2.8.0. Secondly, a normal in-wheel motor and three faulty in-wheel motors operating under the same working conditions are treated as four distinct states of the in-wheel motor, and the training samples for these four states under each working condition are processed collectively. For example, under operating conditions of 100 r/min, there are four states of the in-wheel motor, with 231 training samples for each state; therefore, the total number of training samples for each operating condition is 924. Similarly, 200 test samples and 200 validation samples are obtained for each operating condition. When the training samples for each operating condition are input into the proposed ResNet-KAN, the corresponding diagnostic model completes training. Finally, the recognition accuracy for each state of the in-wheel motor is validated using test samples under the same operating conditions, as shown in Figure 12.

Under identical operating conditions, the ResNet-KAN diagnostic model achieved recognition accuracy exceeding 98% for all states of the in-wheel motor, with particularly outstanding performance in the normal state. At operating speeds of 100, 200, 600 and 700 r/min, the average diagnostic accuracy consistently remained above 99%. In contrast, the accuracy at 400 r/min was relatively lower. This is because the fault characteristic frequency at 400 r/min is close to the natural frequency of the experimental system, resulting in increased resonance-induced noise interference. Consequently, fluctuations in recognition accuracy were observed for all four bearing states at this speed. However, the overall performance still satisfied practical engineering requirements.

To compare the performance of the proposed ResNet-KAN fault diagnosis model with other classical models, this section conducts a statistical analysis of the classification accuracy of each model under identical experimental data conditions. The comparison models include 2D-CNN, ViT and ResNet. The experimental data selected comprise in-wheel motor bearing vibration signals acquired under static operating conditions at a speed of 500 r/min, with all models utilising the same dataset format as the input. To ensure the reliability of the experimental results, each model underwent five independent training and testing cycles.

**Figure 13:**
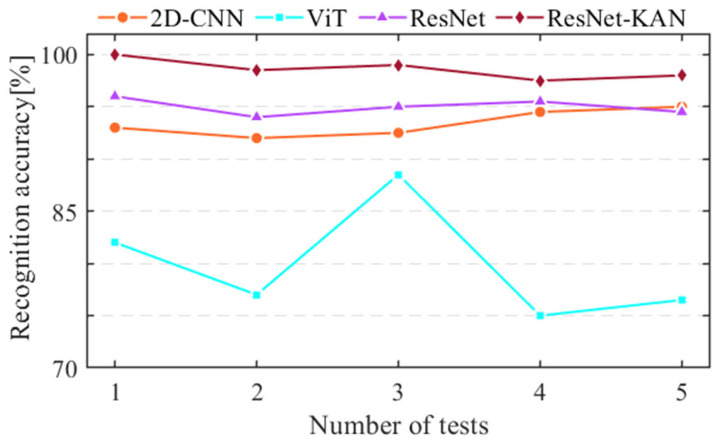
Test accuracy of each fault diagnosis model under the same operating conditions.

As can be seen from Figure 13, the ResNet-KAN model achieved the highest diagnostic accuracy among all compared models, followed by ResNet, whereas the ViT model exhibited relatively poor stability and lower overall accuracy. Specifically, the accuracy of the ResNet-KAN model consistently stood at 98% or above, reaching a maximum of 100%. Compared with the other three methods, its accuracy is at least 2% higher; it consistently maintains a high level of accuracy with minimal fluctuation. The 2D-CNN model demonstrates good stability but has relatively limited accuracy; the ResNet model performs slightly better than the 2D-CNN model in testing, with one test result reaching 95.5%.

#### 4.4.2. t-SNE Visualisation

**Figure 14:**
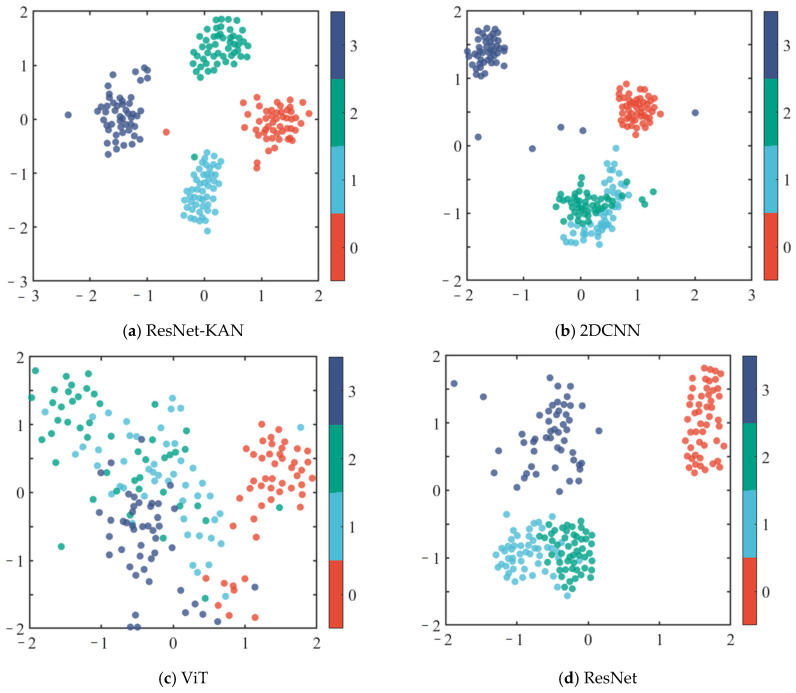
Visualisation of output features for test set samples across different models.

To visually demonstrate the diagnostic performance of the ResNet-KAN model and reveal the differences between features, this chapter employs the t-distributed stochastic neighbour embedding (t-SNE) data dimensionality reduction method proposed by Kerstin [66] to perform dimensionality reduction on the final hidden-layer features from the test set of each model experiment under the same operating conditions, yielding the visualised scatter plots shown in Figure 14.

Figure 14 visualises the output features of the final layer for each model at the same rotational speed. As can be seen from the figure, the ResNet-KAN model demonstrates the best classification performance, with clear differentiation between the three fault states, whilst the ViT model performs poorly, with the normal state and the three fault categories appearing mixed and indistinguishable; for the ResNet-KAN and 2DCNN models, the features for outer-ring and inner-ring faults overlap to some extent, and the differences in features may be significant. However, the ResNet-KAN model demonstrates higher discriminative power between normal and rolling element faults compared to the 2DCNN model. Overall, the ResNet-KAN model exhibits superior performance in both state recognition and fault classification.

#### 4.4.3. Cross-Validation of the Model Under Multiple Working Conditions

The real-world operating conditions of in-wheel motor-driven electric vehicles are highly variable, and model validation under a single operating condition cannot fully demonstrate the superiority of the improved ResNet-KAN model. Therefore, to verify whether the processing performance of the model proposed in this study is significant under multiple operating conditions, a cross-validation scheme for the model under multiple operating conditions was designed.

In this scheme, experimental data from two non-contiguous rotational speeds were used as training samples, whilst data from the intermediate rotational speed were used for testing. For example, experimental data at 100 r/min and 300 r/min were treated as training samples, labelled 100 r/min and 300 r/min, respectively, whilst data at 200 r/min served as test data. During the implementation of the validation scheme, the hyperparameters, learning rate and framework for each model were kept constant. Table 4 shows the recognition accuracy of the cross-validation tests for in-wheel motor bearing conditions at different rotational speeds. Although the theoretical fault frequencies vary with rotational speed, the proposed method focuses on the structural texture characteristics of fault impacts rather than fixed frequency values. After GADF transformation, the vibration signals are encoded into two-dimensional texture patterns, enabling the ResNet-KAN model to maintain stable fault recognition capabilities under different operating conditions without retraining.

Overall, the recognition accuracy of each diagnostic model for the various operating states of the in-wheel motor exceeds 93%, particularly for the normal state of the in-wheel motor, where the recognition accuracy is generally above 95%. Of course, compared with the validation results for the recognition accuracy of the four states of the in-wheel motor under identical operating conditions, the cross-validation accuracy is slightly lower; however, it still meets the required engineering standards. The average accuracy fell below 94% in only one scenario: at rotational speeds of 300 and 500 r/min, which were precisely the speeds used to train the diagnostic models. A comprehensive analysis indicates that, at a rotational speed of 400 r/min, the proximity of the resonance frequency significantly influenced the experimental data.

#### 4.4.4. Ablation Experiment Validation

To further validate the effectiveness of the various architectures within the ResNet-KAN model proposed in this paper, ablation experiments were conducted by removing or replacing specific components of the proposed model. Three schemes were designed for comparison with the method described in this paper.

**Figure 15:**
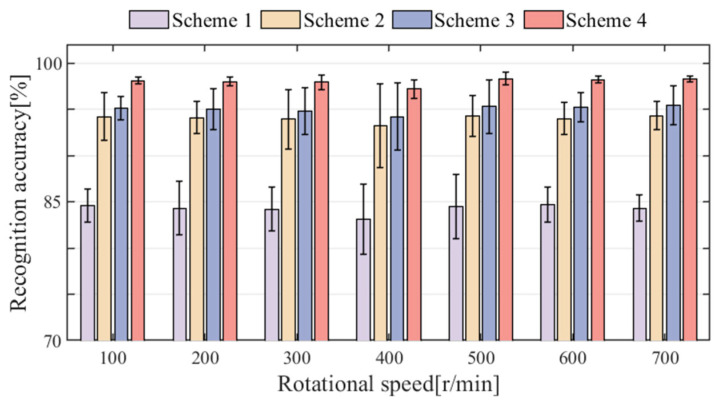
Recognition rates of the ResNet-KAN model and its different ablation experimental schemes.

Scheme 1 removed the GADF preprocessing step and directly used greyscale images as the inputs. Scheme 2 replaced the two ReLU-KAN layers with traditional fully connected layers. Scheme 3 replaced the improved ReLU-KAN with the original B-spline-based KAN structure, while Scheme 4 corresponds to the complete method proposed in this paper. For all schemes, the backbone network structure and training parameters remained unchanged. Each scheme was trained and evaluated using the same dataset under identical operating conditions. The recognition accuracies of the four bearing states for different schemes are presented in Figure 15.

As shown in Figure 15, the proposed ResNet-KAN achieved the best performance in terms of both average accuracy and stability for identifying the four operating states of the in-wheel motor. Specifically, the diagnostic models established based on the proposed method all achieved an accuracy rate exceeding 98.0%, with a maximum variance of less than 0.5%. For Scheme 1 and the method proposed in this paper, the classification accuracy of the ResNet-KAN model without GADF processing was approximately 84.25%, and the variance was significant. This indicates that GADF processing of the data is necessary. For Scheme 2, although the state recognition rate reached approximately 94%, the recognition accuracy remained slightly lower; therefore, the proposed method effectively addresses the limitations of linear mapping inherent in traditional fully connected layers. For Scheme 3, although the average recognition rate exceeds 95%, the maximum variance increases to 1.8%. Consequently, as Scheme 3 employs complex B-spline interpolation functions, it is prone to overfitting with limited samples, resulting in greater fluctuations during cross-condition testing compared to the method proposed in this paper.

#### 4.4.5. Discussion

In response to the difficulty in accurately identifying bearing failure characteristics in in-wheel motors under complex and variable automotive operating conditions, this paper proposes an intelligent fault diagnosis method based on the ResNet-KAN. Firstly, a lightweight ResNet is constructed as the backbone network for feature extraction, utilising residual structures to enhance deep feature representation capabilities and mitigate the issue of gradient vanishing. Building upon this, an improved ReLU-KAN is introduced to replace the traditional fully connected layers, utilising the local basis function modelling capability to enhance the model’s ability to characterise complex non-linear decision boundaries. To validate the superiority of the proposed method, model comparison validation under identical operating conditions, visualisation comparisons, cross-validation across multiple operating conditions, and ablation experiments were conducted, demonstrating the significant advantages of the proposed diagnostic model in in-wheel motor bearing fault diagnosis. Specifically, in the model comparison experiments under identical operating conditions, the ResNet-KAN diagnostic model achieved an average accuracy exceeding 98%, outperforming the 2DCNN model, the ViT model, and the traditional ResNet model; in the cross-validation experiments across multiple operating conditions, the ResNet-KAN diagnostic model achieved an average accuracy exceeding 93.9%. In the ablation experiments, the proposed diagnostic method outperformed all other approaches.

## 5. Conclusions

This paper proposes an integrated intelligent fault diagnosis framework for in-wheel motor bearings under complex operating conditions. The following conclusions are drawn.

Firstly, a CPO-SVMD-based feature extraction method was proposed. The CPO-SVMD method, combined with RE and a multi-strategy optimizer, effectively suppresses strong background noise and adaptively extracts faint impact features. Experimental results confirm its superiority in isolating sensitive fault modes from high-noise vibration signals.

Secondly, a two-dimensional feature representation method based on the GADF was developed. Experimental results demonstrate that the feature extraction achieved by the GADF method significantly enhances the discriminative power of the features; the SSIM values between different states are all kept below 0.11, with an overall mean of just 0.0227.

Finally, an intelligent fault diagnosis model based on ResNet-KAN is proposed. A lightweight ResNet is constructed as the backbone network for feature extraction. In the classification stage, a ReLU-KAN non-linear decision head is introduced to replace the traditional fully connected layers. Experimental results indicate that in comparative tests of different models under identical operating conditions, the proposed method achieves a fault recognition accuracy of over 98%, significantly outperforming the 2DCNN, ViT and traditional ResNet models. Future research will focus on two directions: (1) developing deep transfer learning algorithms to further improve diagnostic reliability under extreme variable operating conditions and cross-load scenarios; (2) optimising the model’s computational efficiency and inference speed to satisfy the real-time requirements of on-board online condition monitoring systems for electric vehicles.

## Figures and Tables

**Table 1:** Optimal penalty factors for the five optimisation algorithms.

Method	PSO	GWO	SSA	CPO
α	2755	1800	1515	1203

**Table 2:** SSIM comparison of different time–frequency analysis methods.

(Time–Frequency Plot a, Time–Frequency Plot b)	STFT	WVD	S-Transform	GADF
(Normal state, outer-race fault)	0.9680	0.7802	0.6844	−0.1102
(Normal condition, inner-race fault)	0.9691	0.7180	0.7321	0.0667
(Normal condition, rolling element fault)	0.9791	0.7687	0.7357	−0.0757
(Outer-race fault, inner-race fault)	0.9541	0.7271	0.6919	0.1119
(Outer-race fault, rolling element fault)	0.9609	0.7849	0.6878	0.0559
(Inner-race fault, rolling element fault)	0.9631	0.7233	0.7424	0.0875
Mean SSIM under the same method	0.9657	0.7504	0.7124	0.0227

**Table 3:** Network configuration parameters of ResNet-KAN.

Network Branch	Main Module	Output Dimension
Initial Convolution Layer	3 × 3 Convolution, BN, ReLU	224 × 224 × 4
Residual Layer 1	Residual block	224 × 224 × 4
Residual Layer 2	Residual block	112 × 112 × 8
Residual Layer 3	Residual block	56 × 56 × 16
Residual Layer 4	Residual block	28 × 28 × 32
Pooling Layer	GAP + GMP	1 × 1 × 32
Constraint Layer	Feature Flattening + Sigmoid Mapping	32
Classification Layer 1	ReLU-KAN	16
Classification Layer 2	ReLU-KAN	4 classes

**Table 4:** Evaluation metrics and recognition accuracy of in-wheel motor bearing status under different rotational speeds.

Rotational Speed of Training Sample	Rotational Speed of Test Sample	Recognition Accuracy for Each Fault Condition				Average Accuracy
		Normal	Inner Race	Outer Race	Rolling Elements	
100 and 300	200	97.9%	94.8%	96.8%	96.5%	96.5%
200 and 400	300	96.5%	92.8%	95.2%	93.6%	94.5%
300 and 500	400	95.0%	93.3%	94.1%	93.2%	93.9%
400 and 600	500	95.6%	94.0%	94.8%	94.4%	94.7%
500 and 700	600	95.2%	93.2%	94.4%	94.0%	94.2%

## Data Availability

The original contributions presented in this study are included in the article. Further inquiries can be directed to the corresponding author(s).

## References

[1] Tao Y., Wang X., Zhang L., Bao X., Xue H., Yue H., Feng H., Yang D. (2025). Fault Diagnosis of In-Wheel Motors Used in Electric Vehicles: State of the Art, Challenges, and Future Directions. Machines.

[2] Yu Y., Hao S., Guo S., Tang Z., Chen S. (2022). Motor torque distribution strategy for different tillage modes of agricultural electric tractors. Agriculture.

[3] Xue H., Song Z., Wu M., Sun N., Wang H. (2022). Intelligent diagnosis based on double-optimized artificial hydrocarbon networks for mechanical faults of in-wheel motor. Sensors.

[4] Wang H., Wang J., Wang X., Lu S., Hu C., Cao W. (2022). Detection and evaluation of the interturn short circuit fault in a BLDC-based hub motor. IEEE Trans. Ind. Electron..

[5] Zhu J., Ouyang X., Jiang Z., Xu Y., Xue H., Yue H., Feng H. (2025). In-wheel motor fault diagnosis method based on two-stream 2DCNNs with DCBA module. Sensors.

[6] Tao L., Liu H., Ning G., Cao W., Huang B., Lu C. (2025). LLM-based framework for bearing fault diagnosis. Mech. Syst. Sig. Process..

[7] Xue H., Liu B., Ding D., Zhou J., Cui X. (2022). Diagnosis method based on hidden Markov model and Weibull mixture model for mechanical faults of in-wheel motors. Meas. Sci. Technol..

[8] Yu S., Pang S., Ning J., Wang M., Song L. (2025). ANC-Net: A novel multi-scale active noise cancellation network for rotating machinery fault diagnosis based on discrete wavelet transform. Expert Syst. Appl..

[9] Sun J., Zhou X., Mao H., Wu X., Zhang X., Li Q. (2017). D iscrimination of pesticide residues in lettuce based on chemical molecular structure coupled with wavelet transform and near infrared hyperspectra. J. Food Process Eng..

[10] Kedadouche M., Liu Z., Vu V.-H. (2016). A new approach based on OMA-empirical wavelet transforms for bearing fault diagnosis. Measurement.

[11] Boudraa A.-O., Cexus J.-C. (2007). EMD-based signal filtering. IEEE Trans. Instrum. Meas..

[12] Xu M., Sun J., Zhou X., Tang N., Shen J., Wu X. (2021). Research on nondestructive identification of grape varieties based on EEMD-DWT and hyperspectral image. J. Food Sci..

[13] Ji K., Li Y., Liu Y., Yu Z., Cheng J. (2024). Vibration signal extraction and analysis of combine harvester based on low-pass filter-eemd combination. Eng. Agrícola.

[14] Dai D., Chen D., Wang S., Li S., Mao X., Zhang B., Wang Z., Ma Z. (2023). Compilation and extrapolation of load spectrum of tractor ground vibration load based on CEEMDAN-POT model. Agriculture.

[15] Dragomiretskiy K., Zosso D. (2013). Variational mode decomposition. IEEE Trans. Signal Process..

[16] Yu Z., Li Y., Du X., Liu Y. (2024). Threshing cylinder unbalance detection using a signal extraction method based on parameter-adaptive variational mode decomposition. Biosyst. Eng..

[17] Nazari M., Sakhaei S.M. (2020). Successive variational mode decomposition. Signal Process..

[18] Wang B., Tang Z., Wang K., Li P. (2024). Failure feature identification of vibrating screen bolts under multiple feature fusion and optimization method. Agriculture.

[19] Sun J., Tian Y., Wu X., Dai C., Lu B. (2020). Nondestructive detection for moisture content in green tea based on dielectric properties and VISSA-GWO-SVR algorithm. J. Food Process. Preserv..

[20] Wang J., Zhang Y., Gu R. (2020). Research status and prospects on plant canopy structure measurement using visual sensors based on three-dimensional reconstruction. Agriculture.

[21] Chang C., Li X., Ju H., Wang G., Duanmu L. (2026). Research on pipeline burst location technology in complex environments based on the CPO-SVMD-MCC method. Measurement.

[22] Abdel-Basset M., Mohamed R., Abouhawwash M. (2024). Crested porcupine optimizer: A new nature-inspired metaheuristic. Knowl.-Based Syst..

[23] Wu H., Li X., Lu H., Tong L., Kang S. (2023). Crop acreage planning for economy-resource-efficiency coordination: Grey information entropy based uncertain model. Agric. Water Manag..

[24] Minhas A.S., Kankar P., Kumar N., Singh S. (2021). Bearing fault detection and recognition methodology based on weighted multiscale entropy approach. Mech. Syst. Signal Process..

[25] Shannon C.E. (1948). A mathematical theory of communication. Bell Syst. Tech. J..

[26] Vollbrecht K.G.H., Wolf M.M. (2002). Conditional entropies and their relation to entanglement criteria. J. Math. Phys..

[27] Pan X., Jiang Y., Li H., Hui X., Xing S. (2024). Numerical simulation of the effect of varying dispersion tooth insertion depth on the jet breakup and hydraulic performance. Biosyst. Eng..

[28] Wang L., Huang T., Cao H.X., Yuan Q.X., Liang Z.P., Liang G.X. (2016). Application of air-assisted liquid-liquid microextraction for determination of some fluoroquinolones in milk powder and egg samples: Comparison with conventional dispersive liquid-liquid microextraction. Food Anal. Methods.

[29] Li Y., Jiang X., Wu J. (2025). Rating entropy and its multivariate version. Mech. Syst. Sig. Process..

[30] Yu S., Fan J., Lu X., Wen W., Shao S., Guo X., Zhao C. (2022). Hyperspectral technique combined with deep learning algorithm for prediction of phenotyping traits in lettuce. Front. Plant Sci..

[31] Chen C., Zhu W., Steibel J., Siegford J., Han J., Norton T. (2020). Classification of drinking and drinker-playing in pigs by a video-based deep learning method. Biosyst. Eng..

[32] He M., He D. (2017). Deep learning based approach for bearing fault diagnosis. IEEE Trans. Ind. Appl..

[33] Liu J., Abbas I., Noor R.S. (2021). Development of deep learning-based variable rate agrochemical spraying system for targeted weeds control in strawberry crop. Agronomy.

[34] Nunekpeku X., Zhang W., Gao J., Adade S.Y.-S.S., Li H., Chen Q. (2025). Gel strength prediction in ultrasonicated chicken mince: Fusing near-infrared and Raman spectroscopy coupled with deep learning LSTM algorithm. Food Control.

[35] Zhang X., Xie H., Zhou Y., Jia L. (2024). Local feature expansion Vision Transformer model for bearing fault diagnosis under noise environments. J. Instrum..

[36] Goodfellow I., Pouget-Abadie J., Mirza M., Xu B., Warde-Farley D., Ozair S., Courville A., Bengio Y. (2020). Generative adversarial networks. Commun. ACM.

[37] Sun J., Cao Y., Zhou X., Wu M., Sun Y., Hu Y. (2021). Detection for lead pollution level of lettuce leaves based on deep belief network combined with hyperspectral image technology. J. Food Saf..

[38] Tian Y., Sun J., Zhou X., Yao K., Tang N. (2022). Detection of soluble solid content in apples based on hyperspectral technology combined with deep learning algorithm. J. Food Process. Preserv..

[39] Li H., Luo X., Haruna S.A., Zareef M., Chen Q., Ding Z., Yan Y. (2023). Au-Ag OHCs-based SERS sensor coupled with deep learning CNN algorithm to quantify thiram and pymetrozine in tea. Food Chem..

[40] Li D., Yang Y., Chang H., Xu Z., Ouyang Q. (2025). Improved ResNet deep learning model-assisted computer vision for intelligent assessment of tencha chlorophyll content during drying. J. Food Compos. Anal..

[41] Li H., Sheng W., Adade S.Y.-S.S., Nunekpeku X., Chen Q. (2024). Investigation of heat-induced pork batter quality detection and change mechanisms using Raman spectroscopy coupled with deep learning algorithms. Food Chem..

[42] Wang H., Fu Z., Lin T., Han C., Zhang W., Song L. (2024). A lightweight improved residual neural network for bearing fault diagnosis. Proc. Inst. Mech. Eng. Part C J. Mech. Eng. Sci..

[43] Guo J., Zhang K., Adade S.Y.S.S., Lin J., Lin H., Chen Q. (2025). Tea grading, blending, and matching based on computer vision and deep learning. J. Sci. Food Agric..

[44] Zhang K., Tang B., Deng L., Liu X. (2021). A hybrid attention improved ResNet based fault diagnosis method of wind turbines gearbox. Measurement.

[45] Xie S., Wang J., Li Y., Yang L. (2025). Bearing fault diagnosis method based on improved meta-ResNet and sample weighting under noise label. Struct. Health Monit..

[46] Wu Z., Jiang H., Liu S., Liu Y., Yang W. (2023). Conditional distribution-guided adversarial transfer learning network with multi-source domains for rolling bearing fault diagnosis. Adv. Eng. Inf..

[47] Wang X., Jiang H., Dong Y., Mu M. (2026). Spatial-channel collaborative multi-scale graph interaction deep transfer learning for unsupervised rotating machinery fault diagnosis. Eng. Appl. Artif. Intell..

[48] Zhao H., Wang B., Fu Y., Li N., Gao Z. (2026). Fixed-time adaptive fault-tolerant control of a multi-mode VTOL UAV with variable prescribed performance boundaries under random disturbances. ISA Transactions.

[49] Zheng T., Wang Q., Shen Y., Lin X. (2022). Gradient rectified parameter unit of the fully connected layer in convolutional neural networks. Knowl.-Based Syst..

[50] Liu Z., Wu J., Cai Y., Wang H., Chen L., Liu Q. (2025). Dual-stage feature specialization network for robust visual object detection in autonomous vehicles. Sci. Rep..

[51] Chang H., Cai J., Ouyang Q. (2025). Intelligent chlorophyll estimation by attention-integrated deep learning and dual-modal fusion in tencha drying using snapshot multispectral camera. J. Sci. Food Agric..

[52] Liu Z., Wang Y., Vaidya S., Ruehle F., Halverson J., Soljačić M., Hou T.Y., Tegmark M. (2024). KAN: Kolmogorov-arnold networks. arXiv.

[53] Zhang L., Fan W., Lu L. (2026). RSS-KAN: Replacing B-splines with ReLU, Sigmoid, and Sine for accurate and efficient Kolmogorov–Arnold Networks. Neurocomputing.

[54] Li Y., Zhang H., Ma S., Cheng G., Yao Q., Zuo C. (2024). A novel method based on stepwise variational modal decomposition and gramian angular difference field for bearing health monitoring. Arab. J. Sci. Eng..

[55] Juan G., Guocheng H., Wei M., Qiao P., Jie M. (2026). Multi-super-synchrosqueezing short-time fractional fourier transform algorithm and its applications. Mech. Syst. Sig. Process..

[56] Yang F., Sun J., Cheng J., Fu L., Wang S., Xu M. (2023). Detection of starch in minced chicken meat based on hyperspectral imaging technique and transfer learning. J. Food Process Eng..

[57] Jia N., Cheng Y., Tian Y., Yang F. (2022). Intelligent fault severity detection of rotating machines based on VMD-WVD and parameter-optimized DBN. Shock Vib..

[58] Prata J.C., da Costa P.M. (2024). Fourier transform infrared spectroscopy use in honey characterization and authentication: A systematic review. ACS Food Sci. Technol..

[59] Zhao Q., Zhao D., Yin W. (2024). EEG emotion recognition based on GADF and AMB-CNN model. Int. J. Numer. Model. Electron. Netw. Devices Fields.

[60] Liu Z., Wang Y., Vaidya S., Ruehle F., Halverson J., Soljacic M., Hou T., Tegmark M. (2025). KAN: Kolmogorov–arnold networks. Proceedings of the International Conference on Learning Representations, 2025.

[61] Bae I., Lee S. (2024). A multi-input convolutional neural network model for electric motor mechanical fault classification using multiple image transformation and merging methods. Machines.

[62] Xian T.J., Morris S., Wai C.K. (2021). Evaluation of worldwide harmonised light vehicles test procedure for electric vehicles using simulation. Int. J. Veh. Perform..

[63] Bakurov I., Buzzelli M., Schettini R., Castelli M., Vanneschi L. (2022). Structural similarity index (SSIM) revisited: A data-driven approach. Expert Syst. Appl..

[64] Al Najjar Y. (2024). Comparative analysis of image quality assessment metrics: MSE, PSNR, SSIM and FSIM. Int. J. Sci. Res. (IJSR).

[65] Gu J., Peng Y., Lu H., Chang X., Chen G. (2022). A novel fault diagnosis method of rotating machinery via VMD, CWT and improved CNN. Measurement.

[66] Chen W., Wang H., Zhang Y., Deng P., Luo Z., Li T. (2010). T-distributed stochastic neighbor embedding for co-representation learning. ACM Trans. Intell. Syst. Technol..

